# Comparative Transcriptome Profiling of Rice Near-Isogenic Line Carrying *Xa23* under Infection of *Xanthomonas oryzae* pv. *oryzae*

**DOI:** 10.3390/ijms19030717

**Published:** 2018-03-02

**Authors:** Rezwan Tariq, Chunlian Wang, Tengfei Qin, Feifei Xu, Yongchao Tang, Ying Gao, Zhiyuan Ji, Kaijun Zhao

**Affiliations:** National Key Facility for Crop Gene Resources and Genetic Improvement (NFCRI), Institute of Crop Sciences, Chinese Academy of Agriculture Sciences (CAAS), Beijing 100081, China; rj.rezwan@gmail.com (R.T.); Wangchunlian@caas.cn (C.W.); qintengfeisam@163.com (T.Q.); xvfeifei61@163.com (F.X.); tangyongchao281@126.com (Y.T.); gaoying@caas.cn (Y.G.); jizhiyuan@caas.cn (Z.J.)

**Keywords:** bacterial blight, RNA-Seq, rice, *Xa23*, differentially expressed genes

## Abstract

Bacterial blight, caused by *Xanthomonas oryzae* pv. *oryzae* (*Xoo*), is an overwhelming disease in rice-growing regions worldwide. Our previous studies revealed that the executor *R* gene *Xa23* confers broad-spectrum disease resistance to all naturally occurring biotypes of *Xoo*. In this study, comparative transcriptomic profiling of two near-isogenic lines (NILs), CBB23 (harboring *Xa23*) and JG30 (without *Xa23*), before and after infection of the *Xoo* strain, PXO99^A^, was done by RNA sequencing, to identify genes associated with the resistance. After high throughput sequencing, 1645 differentially expressed genes (DEGs) were identified between CBB23 and JG30 at different time points. Gene Ontlogy (GO) analysis categorized the DEGs into biological process, molecular function, and cellular component. KEGG analysis categorized the DEGs into different pathways, and phenylpropanoid biosynthesis was the most prominent pathway, followed by biosynthesis of plant hormones, flavonoid biosynthesis, and glycolysis/gluconeogenesis. Further analysis led to the identification of differentially expressed transcription factors (TFs) and different kinase responsive genes in CBB23, than that in JG30. Besides TFs and kinase responsive genes, DEGs related to ethylene, jasmonic acid, and secondary metabolites were also identified in both genotypes after PXO99^A^ infection. The data of DEGs are a precious resource for further clarifying the network of *Xa23*-mediated resistance.

## 1. Introduction

Pathogens cause extensive damage to agriculture and food commodities. According to molecular clocks, land plants evolved 700 million years ago, and the establishment of land plants was facilitated by the symbiotic microbe association [1]. Interestingly, the epidemiological plant–microbe interaction provides the co-evolutionary arms race that varies in both time and space. For example, the increased virulence of a pathogen places strong selection on plants to modify their defense response. If successful resistance is developed, selection pressure is exerted on the pathogen for changing its tactics to overcome the developed resistance [2,3].

Rice is an important cereal crop which influences the economies of several billion people, especially in Asia, Latin America, and the Middle East. In rice, more than 70 diseases instigated by bacteria, fungi, nematodes, and viruses are prevalent [4]. Among bacterial diseases, bacterial blight, caused by *Xanthomonas oryzae* pv. *oryzae* (*Xoo*), is an overwhelming disease in most of the rice growing regions worldwide [5]. Bacterial blight was first reported by the Japanese farmers in 1884, but not taken seriously due to it causing less damage [6]. However, this disease appeared again drastically in high yielding varieties during 1960–1970 [7]. Since then, the infection of the *Xoo* has been widely reported throughout Asia, and become one of the serious rice diseases, bringing about dreadful yield loss [8]. Typically, *Xoo* is a vascular pathogen, and enters the host through hydathodes of leaf margins and wounds, multiplies into the intercellular spaces beneath epithelial tissues, then moves to the xylem vessels for systemic infection [9]. During infection, *Xoo* injects transcription activator like-effector (TALE) protein into the host cell via the type III secretion system that activates the susceptible gene to promote the disease progression, or may activate the resistance gene that triggers the defense mechanism [10,11]. Interestingly, natural TALEs have central conserved repeat region of 34-amino acid-repeats (34-aa-repeats), an N terminus region for type III secretion system (TTSS), C-terminus containing acidic transcription activation domain (AD), and nuclear localization signal (NLS). Moreover, the central repeat region (CRR) of TAL effectors determines the targeted genes in the host plant cells [10].

Until now, 42 resistance genes have been identified to tackle the increasing threat of bacterial blight [12,13]. *Xa23*, a dominate bacterial blight resistance gene, confers broad-spectrum resistance against all naturally occurring biotypes of *Xoo* [14]. The expression of *Xa23* causes hypersensitivity response (HR) or programmed cell death (PCD) in plants, which ultimately restricts the pathogen infection in disease resistant plants. Basically, *Xa23* was derived from a wild race of rice (*Oryza rufipogon*) that confers broad-spectrum resistance against *Xoo*. A near-isogenic line (NIL), CBB23, was developed from a cross between *O*. *rufipogon* and susceptible *indica* variety JG30 [15].

Because *Xa23* confers broad-spectrum resistance against *Xoo*, it is necessary to conduct an independent study to compare the gene expression profiles of the CBB23 and JG30 rice genotypes during different *Xoo* infection stages (12, 24, 36, and 48 h of post inoculation (hpi)). For comparative analysis, RNA-Seq allows transcriptomic analysis in an unbiased way, a tremendous detection range (>8000-fold) with single base pair resolution and low background signals [16]. Moreover, RNA-Seq has an ability to detect the differentially expressed genes with a broader dynamic range of expression levels [17]. Several transcriptomic profiling studies of plants, e.g., wheat, maize, rice, *Arabidopsis*, and watermelon, etc., under different biotic and abiotic stresses, have been investigated successfully [18,19,20,21]. Here we conducted a comparative transcriptomic study to identify the differentially expressed genes (DEGs) in both NILs (CBB23 and JG30) after PXO99^A^ infection at the early and late time period. After transcriptomic analysis, we identified several DEGs, including different transcription factors (TFs), kinase responsive genes, and clusters of genes involved in different gene ontology terms (GO) and Kyoto encyclopedia of genes and genomes (KEGG) pathways. These DEGs would be a valuable resource for future molecular studies of rice resistance against *Xoo* infection.

## 2. Results

### 2.1. RNA Sequencing of Different Samples and Data Analysis

**Figure 1:**
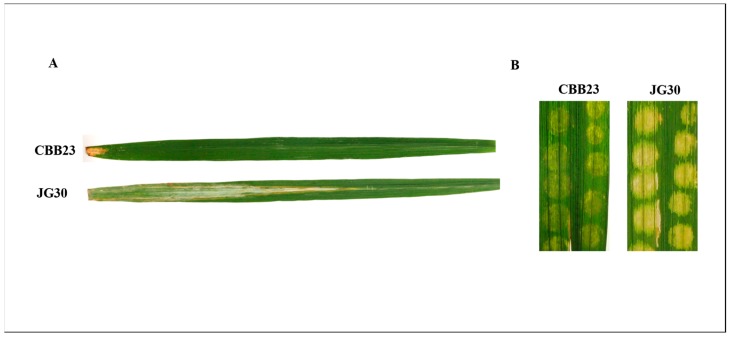
Reaction patterns of CBB23 and JG30 leaves to *Xoo* strain PXO99^A^. (**A**) Comparison of CBB23 and JG30 leaves after inoculation of PXO99^A^ strain by scissors-dipped method. Photographs were taken 14 days post inoculation; (**B**) Inoculation of PXO99^A^ strain into CBB23 and JG30 leaves with a needleless syringe. Photographs were taken 4 days post inoculation.

First, leaves of CBB23 and JG30 genotypes were infected by PXO99^A^ via scissors-clipping method, for the confirmation of resistant and susceptible genotypes (Figure 1A). To investigate the comparative expression of genes in the NILs of rice, RNA was extracted from mock (C0 and J0) as well as leaves inoculated with PXO99^A^ (12 hpi, 24 hpi, 36 hpi, and 48 hpi), and cDNA libraries were prepared for RNA-Seq (Figure 1B). Additionally, raw reads were ranged from 41893788 to 65574380 (Table 1 and Table S1). After a quality check, adapter and low-quality reads were eliminated from data. The clean data were ranged from 40,539,554 to 62,882,740 reads. The sequence reads were mapped to the rice reference genome using TopHat and implementing Bowtie. The number of reads, ranged from 79.99% to 83.96%, were mapped to the rice reference genome. Furthermore, GC contents were ranged from 52.85% to 55.23%. The mapping data depicted that >80% clean reads were successfully mapped to the rice reference genome (maximum of two mismatches). The high range of genome coverage of our RNA-Seq data revealed that DEGs data were reliable for further bioinformatics analysis.

### 2.2. Identification of DEGs in Response to PXO99^A^ in CBB23 and JG30

**Figure 2:**
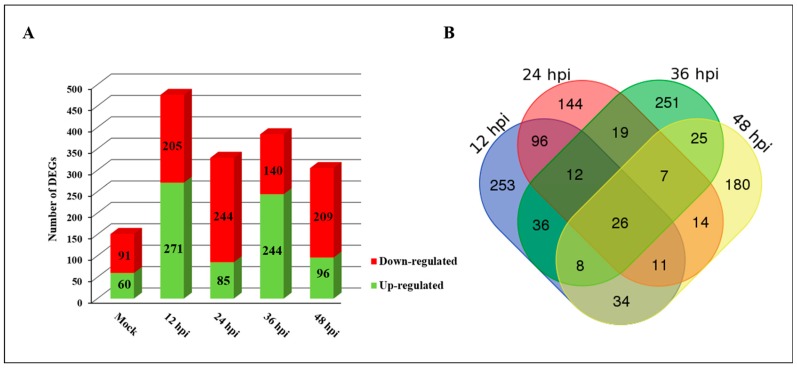
Differentially expressed genes retrieved from CBB23 compared to JG30 at different time of intervals. (**A**) Overall, 1645 DEGs were perceived in CBB23 compared to JG30 at different time of intervals after PXO99**^A^** infection; (**B**) Venn diagram illustrating the differentially expressed genes at 4 different times of interval after PXO99^A^ infection in CBB23 compared with JG30. A set of 476, 329, 384, and 305 DEGs were identified at 12 hpi, 24 hpi, 36 hpi, and 48 hpi, respectively.

To understand the expression pattern of genes induced by PXO99^A^ in both resistant (CBB23) and susceptible (JG30) genotypes, pairwise comparisons were made between CBB23 vs. JG30 at a specific time interval. A *p*-value ≤ 0.05 threshold was set to retrieve the significantly DEGs in CBB23 and JG30 at different time intervals before and after PXO99^A^ infection. Moreover, DEGs were screened on the basis of log2fold change (log_2_FC) ≥1 or ≤−1. Merely, DEGs identified by *p*-value ≤ 0.05 and log_2_FC ≥1 or ≤−1 were kept for further analysis (Table S2); a total of 1645 DEGs were identified between CBB23 and JG30 before and after PXO99^A^ infection (Figure 2A). The comparison between two genotypes revealed that there were more downregulated genes than upregulated genes. Most of the genes were upregulated during 12 hpi and 36 hpi of PXO99^A^. Concisely, we identified 151 (60 upregulated and 91 downregulated), 476 (271 upregulated and 205 downregulated), 329 (85 upregulated and 244 downregulated), 384 (244 upregulated and 140 downregulated), and 305 (96 upregulated and 209 downregulated) in mock (C0 vs. J0), 12 hpi, 24 hpi, 36 hpi, and 48 hpi, respectively. Furthermore, the DEGs overlapped in CBB23 vs. JG30 were analyzed; though 26 DEGs were overlapped in all time points, 253, 144, 251, and 180 DEGs were distinctive at 12 hpi, 24 hpi, 36 hpi, and 48 hpi, respectively (Figure 2B).

### 2.3. Response of Differentially Expressed Transcription Factors to PXO99^A^ Infection

**Figure 3:**
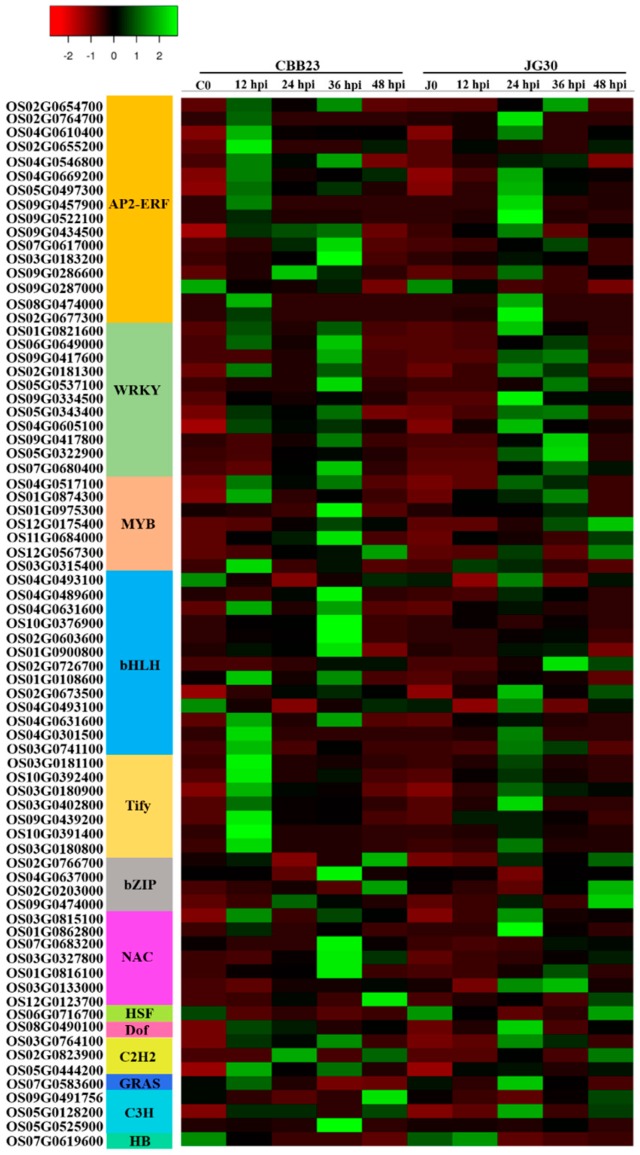
Heat maps exhibiting the FPKM (fragments per kilobase pair of exon model per million fragments mapped) based expression pattern of differentially expressed transcription factors at different time points in CBB23 and JG30 before and after the PXO99^A^ inoculation. The data of TFs were retrieved from Plant TFDB (Plant transcription factor database) and cross-checked with RAP-DB.

Transcription factors appear to be involved in the regulation of different physiological programs in response to plant–pathogen interaction [22]. We identified 75 putative transcription factors that could be classified into 13 different TF families (Figure 3 and Table S3). These 75 differentially expressed TFs might perform an important role in the rice–PXO99^A^ interaction. Among differentially expressed TFs, only 3 WRKY TFs (Os09G0417600, Os09G0417800, and Os05G0322900) were upregulated in C0 vs. J0; whereas, nine APETALA2/ethylene responsive factor (AP2-ERF), seven WRKY, two MYB, three bHLH, and seven Tify TFs were upregulated in CBB23 relative to JG30 at 12 hpi. At 24 hpi, eight AP2-ERF, six WRKY, five bHLH, and five Tify were the major downregulated TFs; however, only one C2H2 TF was upregulated in CBB23 compared to JG30 at 24 hpi. At 36 hpi, five AP2-ERF, seven bHLH, and three NAC TFs were the major genes that had high expression patterns in CBB23 relative to JG30. Moreover, one C3H and one NAC TFs were upregulated in CBB23 compared to JG30 at 48 hpi. Briefly, 47 TF responsive genes were upregulated and 28 were downregulated in CBB23 relative to JG30. Thus, upregulated genes may be involved in enhancing the immunity of CBB23 against PXO99^A^ infection. Nevertheless, downregulated genes may be negatively regulating the rice immunity upon PXO99^A^ infection.

### 2.4. Response of Different Kinase Responsive Genes to PXO99^A^ Infection

**Figure 4:**
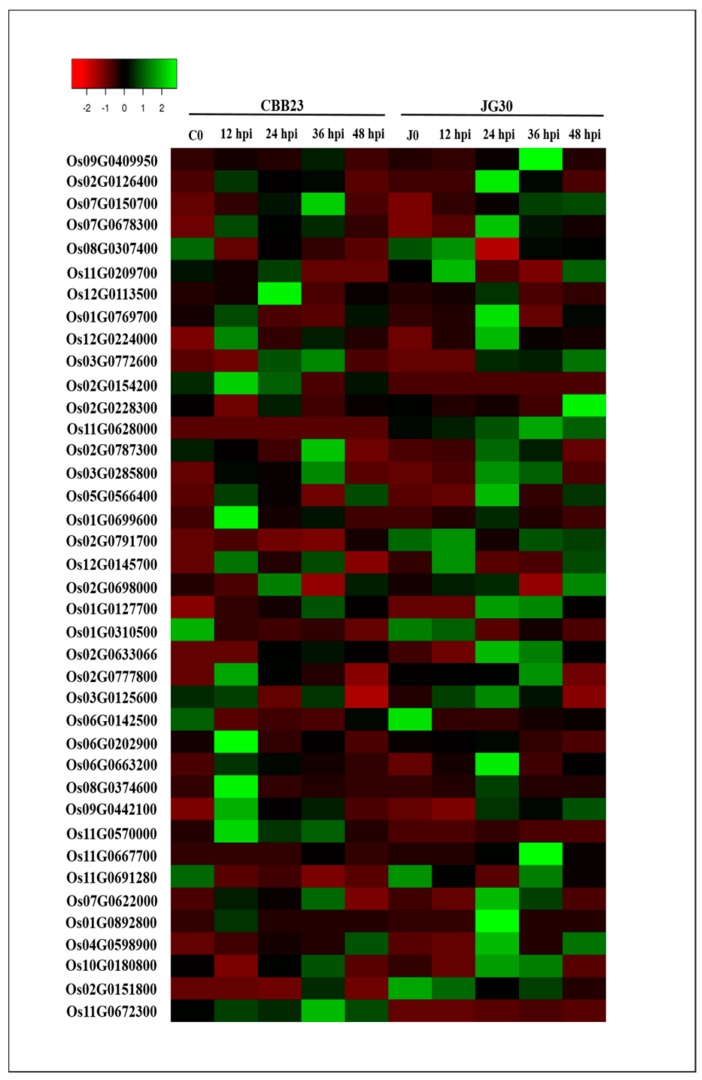
Heat maps illustrating the FPKM based expression pattern of different kinase responsive genes in CBB23 and JG30 before and after PXO99^A^ inoculation at different time points. The data of kinase responsive genes were retrieved from RAP-DB and rice kinase database (RKD).

Kinases play a crucial role in signaling during pathogen recognition and subsequent activation of the plant defense mechanism to counter the pathogen attack. In our experiments, we identified 39 differentially expressed kinase responsive genes at different time points before and after the PXO99^A^ infection (Figure 4). Among the 39 kinase responsive genes, expressions of 13 genes were upregulated, and 26 were downregulated in CBB23 relative to JG30 after PXO99^A^ infection (Table S4). In mock samples (C0 vs. J0), expression of three genes (Os11G0628000, Os02G0151800, and Os02G0791700) were downregulated, and only one gene (Os11G0672300) was upregulated. At 12 hpi, 23 DEGs (13 upregulated and 10 downregulated) were identified. Among the 13 upregulated DEGs, including three receptor like kinases (RLKs) (Os09G0442100, Os08G0374600, and Os11G0570000), 3 serine/threonine protein kinase (Os01G0699600, Os06G0202900, and Os07G0622000), 2 mitogen-activated protein kinase (MAPK) (Os03G0285800, Os05G0566400), one calcium-dependent protein kinase (Os02G0126400), one phytosulfokine receptor (PSK) (Os02G0154200), one ankyrin-kinase (Os01G0892800), one CBL-interacting protein kinase (Os07G0678300), and one kinase responsive protein (Os11G0672300), all exhibited positive regulation in CBB23 genotype compared to the JG30 genotype. At 24 hpi, 20 DEGs (3 upregulated and 17 downregulated) were identified. Among the 20 DEGs, three differentially expressed kinase responsive genes, including one CBL-interacting protein kinase (Os12G0113500), one phosphatidylinositol 3-kinase (Os08G0307400), and one kinase responsive protein (Os11G0672300), were upregulated in CBB23 than in JG30. Differentially expressed kinase responsive genes with seven downregulated genes at 36 hpi; however, one DEG belong to the MAPK (Os02G0787300), one kinase responsive protein (Os11G0672300), respectively, were upregulated in CBB23 relative to JG30. At 48 hpi, eight DEGs were identified, and the expression of one kinase responsive protein (Os11G0672300) was upregulated, and the other seven genes were downregulated in CBB23 vs. JG30 after PXO99^A^ infection.

### 2.5. GO Based Analysis of the DEGs

The gene ontology (GO) analysis was done by using AgriGO online tool. The DEGs were categorized into three different GO categories, i.e., (1) biological process, (2) molecular function, and (3) cellular component. The detailed GO analysis of 1645 DEGs between CBB23 vs. JG30 is shown in Table S5. To investigate the effect of PXO99^A^ on the expression of the genes in rice, we analyzed the DEGs at different time intervals in terms of GO. Moreover, significantly enriched GO terms were retrieved by using the false discovery rate (FDR) ≤ 0.05 (Table S6). In C0 vs. J0, DEGs were grouped into 13 different GO terms (FDR ≤ 0.05), including 11 cellular components and two molecular function related terms. In CBB23-12 hpi vs. JG30-12 hpi, we identified 19 different GO terms (FDR ≤ 0.05), and the major terms were covered by molecular function (17) and cellular component (2). In CBB23-24 hpi vs. JG30-24 hpi, the DEGs were significantly enriched in biological process (17) followed by cellular component (18) and molecular function (5) related different GO terms (FDR ≤ 0.05). In CBB23-36 hpi vs. JG30-36 hpi, the significantly enriched 42 different GO terms (FDR ≤ 0.05) were classified into biological process (14), cellular component (16), and molecular function (12). In CBB23-48 hpi vs. JG30-48 hpi, the DEGs were remarkably enriched in 41 GO terms (FDR ≤ 0.05) consisting of biological process (15), cellular component (19), and molecular function (7). 

**Figure 5:**
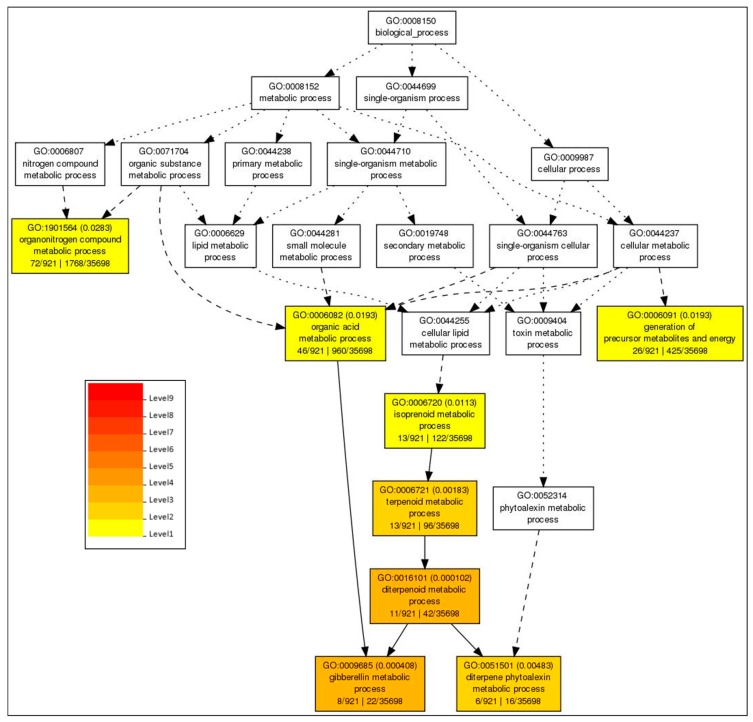
Enriched biological process related GO terms after PXO99^A^ infection. The boxes in the graphs are marked with their GO terms. The different colors schemes mention the significance level of GO terms.

To identify which biological process was differentially regulated after PXO99^A^ inoculation, the GO analysis for the classification of 12 hpi, 24 hpi, 36 hpi, and 48 hpi DEGs was performed (Figure 5). The GO terms revealing the biological process were as follows: “organonitrogen compound metabolic process” (GO:1901564), “organic acid metabolic process” (GO:0006082), “generation of precursor metabolites and energy” (GO:0006091), “isoprenoid metabolic process” (GO:0006720), “terpenoid metabolic process” (GO:0006721), “diterpenoid metabolic process” (GO:0016101), “gibberellin metabolic process” (GO:0009685), and “diterpene phytoalexin metabolic process” (GO:0051501).

### 2.6. MapMan Overview of DEGs Related to Plant–Pathogen Interaction

**Figure 6:**
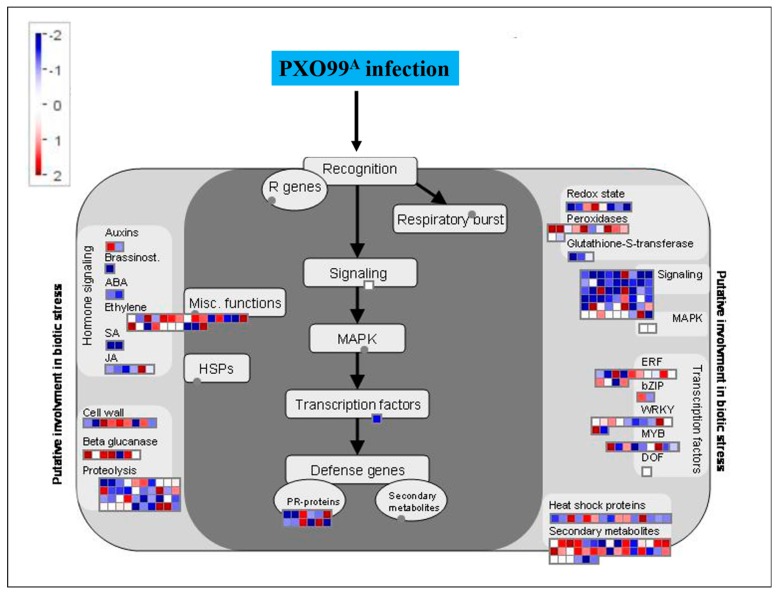
MapMan visualization of defense response in CBB23 relative to JG30 after PXO99^A^ infection. DEGs (*p*-value ≤ 0.05) were imported to MapMan for visualization. The range for the indication of up and downregulated genes is shown in red and blue colors, respectively. The log_2_FC are shown in scale bar.

To elucidate the role of PXO99^A^ in the induction of DEGs in rice, the DEGs that had a role in defense during the plant-pathogen interaction were retrieved. For this, MapMan package was employed to investigate the pathway between rice-PXO99^A^ interactions. Basically, MapMan package uses the input data of DEGs to form a particular biological process by the using available rice genome database. An overview of the regulation of DEGs, such as TFs, kinase responsive genes, PRs, and peroxidases, etc., is shown in Figure 6. Most of the DEGs belonging to ERFs were upregulated, while most of the DEGs encoding WRKY, MYB, and Dof-related DEGs were downregulated. Four of 12 DEGs encoding PRs were upregulated, and the other eight DEGs were downregulated. Twenty-one of 36 DEGs associated with secondary metabolites were upregulated, and other 15 DEGs were downregulated. Expression of 56 DEGs associated with signaling was influenced by PXO99^A^ in CBB23 vs. JG30. Among the 56 DEGs, 38 genes were downregulated, and 18 signaling-associated genes were upregulated. Eight of the 12 DEGs associated with peroxidase and 3 DEGs from glutathione *S*-transferase were downregulated.

After PXO99^A^ infection, two DEGs encoding abscisic acid (ABA) and two DEGs related to the salicylic acid (SA) were downregulated in CBB23 relative to JG30.

Fourteen of 25 DEGs encoding ethylene (ET) were upregulated, and five DEGs associated with cell wall were upregulated. In addition, five of seven DEGs were upregulated in beta-glucanase, and 13 DEGs encoding proteolysis were upregulated. The detailed list of all the DEGs involved in rice–PXO99^A^ interaction in MapMan functional categories was pointed out in Table S7.

### 2.7. KEGG Pathway

To perform the pathway analysis of the 1645 DEGs between CBB23 and JG30 at different time intervals before and after the inoculation of PXO99^A^, we mapped the DEGs in the KEGG database. The KEGG pathways were scrutinized on the basis of *p*-value ≤ 0.05 (Table S8). The “biosynthesis of phenylpropanoids” and “biosynthesis of plant hormones” were prominent pathways between CBB23 and JG30 in response to PXO99^A^ at 12, 24, 36, and 48 hpi.

**Figure 7:**
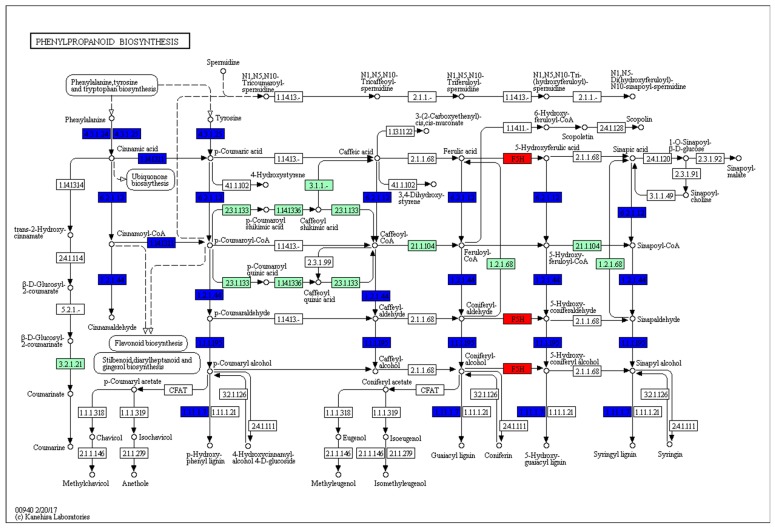
Putative phenylpropanoid biosynthesis pathway was constructed based on the log_2_FC value of DEGs. The number in boxes represents the enzymes coding. The different color schemes were given to the boxes: blue and red colors depict the up- and downregulated genes, respectively.

“Biosynthesis of phenylpropanoids” and “flavonoid biosynthesis” were enriched at 24 and 36 hpi. Besides these above-mentioned pathways, “glycolysis/gluconeogenesis” was enriched at 36 and 48 hpi. In detail, as a result of PXO99^A^ infection, the sketch of phenylpropanoid pathway revealed the most relevant biological function of the DEGs (Figure 7). The upregulated DEGs were in phenylalanine ammonia-lyase (EC:4.3.1.24), phenylalanine ammonia-lyase (EC:4.3.1.25), cinnamate 4-hydroxylase CYP73 (EC:1.14.13.11), 4-coumarate-CoA ligase 1 (EC 6.2.1.12), cinnamoyl CoA reductase (1.2.1.44), mannitol dehydrogenase (EC:1.1.1.195), and 1-cys peroxiredoxin (EC:1.11.1.7); while DEGs involved in aldehyde 5-hydroxylase (EC:1.14.-.-) were downregulated. The above results suggest that these pathways may perform to enhance the rice immunity against the PXO99^A^.

### 2.8. Validation of RNA-Seq Results by qRT-PCR

**Figure 8:**
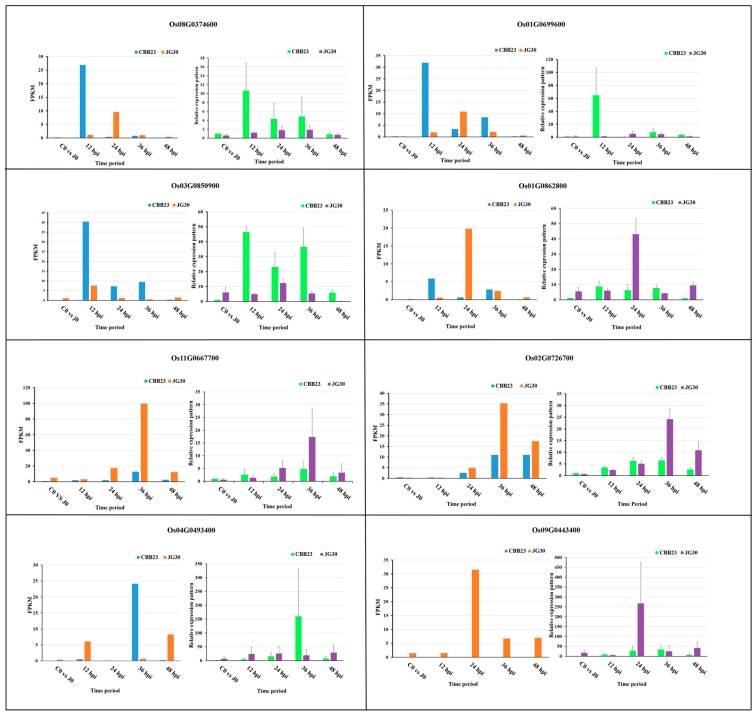
qRT-PCR based validation of DEGs in response to PXO99^A^ at different time intervals. The DEGs were randomly selected for qRT-PCR; the data were normalized by using ubiquitin as an internal reference. Data are represented as mean ± SD for three biological replicates.

Eight DEGs genes influenced by PXO99^A^ between CBB23 and JG30 were selected for qRT-PCR to validate the RNA-Seq data (Figure 8). Among the eight selected genes, Os08G0374600 (receptor kinase CRINKLY4) and Os01G0699600 (serine/threonine protein kinase) were highly expressed at 12 hpi in CBB23 than JG30. Os03G0850900 encoding another kinase exhibited continuous high expression patterns from 12 to 36 hpi in CBB23 compared with JG30. Os01G0862800, responsible for *OsNAC59*, showed a higher expression pattern at 24 hpi in JG30 than CBB23; hence, *OsNAC59* was identified to be important for susceptibility. Os11G0667700 was identified as a kinase responsive gene, and exhibited high expression level in JG30 compared with CBB23 at 36 hpi. *OsbHLH34* (Os02G0726700) was upregulated at 36 hpi and 48 hpi in JG30 compared to CBB23. Os04G0493400 encoding a pathogenesis-related protein (PR) expressed more highly in CBB23 than JG30 at 36 hpi. Os09G0443400 encoding a hypothetical protein exhibited significantly higher expression level at 24 hpi in JG30 than CBB23. In short, the expression pattern of DEGs by qRT-PCR validates the results revealed by RNA-Seq analysis.

## 3. Discussion

In our ecosystem, plants are surrounded by different microorganisms, e.g., bacteria, fungi, viruses, and nematodes. Bacteria become pathogens when they invade the host cell to proliferate [23]. Damaging is a crucial process for pathogens to get the nutrients from plant cell for their survival. The main nutritional component is sugar, located in the apoplast of the cell, but abundant amount is present in cellular matrix. Successful penetration to approach for the cellular matrix is not an easy job for the pathogen. To tackle the invading pathogen, plants have two layers of defense: the pathogen-associated molecular patterns (PAMP)-triggered immunity (PTI) and effector-triggered immunity (ETI).

Usually, PTI is the first line of defense to counter the pathogen attack by recognizing the PAMPs through pattern recognition receptors (PPRs). ETI is a system that recognizes pathogen effectors and induces local cell death, often referred to as hypersensitivity reaction (HR) in plants [24]. NB-LRRs are proteins encoded by *R* genes and present a leucine-rich repeat (LRR), giving them specificity for binding interactions. Race-specific interactions between *Xoo* and rice are thought to follow the gene-for-gene model, which predicts that incompatible interactions are the result of positive function conferred by avirulence genes in the pathogen, and corresponding resistance *R* genes in the host [25]. *Xa23*, an executor R gene, confers a broad spectrum resistance against bacterial blight because AvrXa23 is widespread in all naturally occurring *Xoo* strains [14]. 

Through RNA-Seq, the NILs (CBB23 and JG30) were exploited to identify the defense response in the form of DEGs towards PXO99^A^. A clear morphological distinction between the leaves of CBB23 and JG30 was observed after 10 days of the PXO99^A^ inoculation. *Xa23*-mediated resistance was observed in the form of HR reaction in CBB23; while clear PXO99^A^ growth in form of chlorotic leaf was observed in JG30 leaves. Genome-wide profiles of the gene expression and related pathways differed significantly between these two NILs after PXO99^A^ infection. DEGs upregulated in CBB23 were downregulated in susceptible JG30, at different time intervals of PXO99^A^ post inoculation.

GO analysis identified the high proportion of the genes related to the intracellular organelle, organelle, intracellular membrane-bounded organelle, membrane-bounded organelle, intracellular part, cell part, and cell. Most of the DEGs belonging to the above-mentioned GO terms were also expressed before PXO99^A^ infection in CBB23 vs. JG30. DEGs related to terpenoid metabolic process (GO:0006721), diterpenoid metabolic process (GO:0016101), and diterpene phytoalexin metabolic process (GO:0051501) were significantly expressed in CBB23 compared to JG30 after PXO99^A^ infection. Previous studies approved that rice produces diterpenoids and phytoalexins that exhibit activity against *Xoo* [26]. Furthermore, it was found that terpenoids and phytoalexins induced by pathogens in rice showed antibiotic activities against *Magnaporthe oryzae* [27]. Calcium ion-binding protein (GO:0005509)-related genes were differentially expressed in CBB23 relative to the JG30 that may be involved in plant defense against pathogen attack; for example, *OsCPK10* and *OsCPK18* were observed to be positive and negative regulators of *M. oryzae* resistance, respectively [28,29].

Moreover, to boost the innate immune response, plants produce different reactive oxygen species (ROS) to restrict the pathogen entry into the plant cell through strengthening cell wall and callose deposition. Peroxidases (Prxs) create a physical barrier to confine the pathogen entry into the plant tissues by catalyzing the crosslinking of cell components [30]. DEGs belonging to the peroxidase GO term (GO:0004601) were upregulated in CBB23 after PXO99^A^ infection, compared with JG30. The DEGs encoding peroxidase activity (GO:0004601) were as follows: Os04G0688100, Os07G0677100, Os07G0677500, Os01G0963000, Os12G0448900, Os04G0688300, Os03G0235000, Os06G0547400, Os10G0109600, Os07G0677200, and Os02G0236800. In a previous study, a thylakoid membrane-bound ascorbate peroxidase, OsAPX8, played role in enhancing plant resistance to *Xoo* [31]. Comparative transcriptomic analysis of rice stripe virus and small brown plant hoppers-infected rice leaves during early infection revealed that 12 genes encoding peroxidase class III were significantly upregulated in resistant cultivar to inhibit the infection [32]. In wheat, levels of ROS species were increased in root tissues after *Rhizoctonia* *solani* infection [33]. It was proposed that Prxs interaction with extensin (Tyr residues) makes the cell wall too hard for pathogens to penetrate [34]. Besides the Prxs interaction with Tyr extensin, pectin could act as an anchor for Prxs; the crosslinking between Prxs and pectin creates a dense and solid host plant cell wall, with the aim of limiting the pathogen entry into the plant cell [35,36].

Plant protein kinases are involved to regulate and mediate the different signal transduction processes during abiotic and biotic stresses. In our study, phytosulfokine receptor genes (PSKs), Os02G0154200 were more upregulated in CBB23 than JG30 at 12 hpi. PSKs are the intercellular signals that induce the cellular dedifferentiation at a low cellular level in *Arabidopsis*. Moreover, PSKs are the ligands of LRR-LRKs, crucial for regulating the plant–pathogen interaction, including plant innate immunity [37,38]. The serine/threonine encoding genes, i.e., Os01G0699600 and Os07G0622000, were upregulated at 12 hpi in CBB23 vs. JG30. PTO protein, a serine/threonine kinase, is predicted to be responsible for programmed cell death (PCD) against *Pseudomonas syringae* pv. *tomato* [39]. It is proposed that *Pto* encodes serine/threonine kinase gene has resistance to *Pseudomonas syringae* in tomato [40]. We identified three different genes (Os08G0374600, Os09G0442100, Os11G0570000) belonging to receptor-like kinase (RLKs). After infection of PXO99^A^, identified RLKs were upregulated at 12 hpi in CBB23 vs. JG30. Especially in rice, RLKs were predicted to be involved in response to various abiotic and biotic stresses [41]. An RLKs gene, *Xa26*, was induced by *Xoo*, and played a crucial role in resistance against bacterial blight [42]. In short, RLKs regulate the plant immune response to *Xoo* and *M. oryzae* in rice, and *P. syringae* pv. *tomato* (*Pst*) DC3000 in *Arabidopsis*, respectively [43]. *RLK185* and *RLK55* are positive regulators of plant immune response triggered by chitin and peptidoglycan [44]. In wheat, different RLKs seemed to be upregulated in WL711 + Lr57 (resistance) than WL711 (susceptible) after leaf rust disease [20].

Similarly, single immune receptor activation triggers various immune responses when plants face different biotic stresses in natural environments. Particularly, plants coordinate their stress responses with growth to maximize their fitness. Recent studies depicted that signal transduction is controlled by different TF regulatory networks. Different TFs, i.e., WRKY, NAC, bZIP, and AP2/ERF, etc., have been identified to be differentially expressed in CBB23 after PXO99^A^ infection compared with JG30. WRKY TFs, i.e., *OsWRKY28*, *OsWRKY76*, *OsWRKY7*, and *OsWRKY53* were induced after PXO99^A^ in CBB23 vs. JG30. Previous reports suggested that *OsWRKY28* was upregulated by the infection of *M. oryzae* in rice [45,46]. *OsWRKY76* is responsible for activation of a pathogenesis-related gene, *OsPR10*, contributing to the resistance against *Xoo* [47]. In *Arabidopsis*, *AtWRKY7* is induced by biotic stress and involved in plant defense [48]; moreover, *OsWRKY53* was upregulated in resistant rice cultivar after infection by three different strains of *M. oryzae* (BR29, BR32, and FR13) [46,49].

AP2/ERF TFs, found only in plants, are involved in the regulation of various disease resistance pathways [50]. Though *OPBP1* is a tobacco gene, it enhances the resistance to pathogen when expressed ectopically in transgenic rice [51]. AP2/ERF were suggested to be involved in the signaling of ethylene/jasmonic acid (ET/JA) and regulation of some PR genes; the transcription level of *OsAP2* is significantly increased by SA, H_2_O_2_, and JA [52]. *OsERF922* negatively regulates the resistance to *M. oryzae* by suppressing the expression of the genes encoding phytoalexin biosynthetic enzymes [53].

The expression of MYB-related TFs was observed after PXO99^A^ infection in CBB23 vs. JG30; *OsMYB4* (Os04G0517100) and *R2R3-MYB* (Os01G0874300 and Os12G0567300) were observed to be upregulated in CBB23 compared with JG30 after PXO99^A^ infection. *MYB4* was proposed to mediate the sheath blight resistance by binding to the promoter of peroxidase and oxidoreductase responsive genes [54]. R2R3-MYB TF controls a wide variety of processes, including phenylpropanoid metabolism and secondary cell wall formation [55]. Cell wall formation is a crucial event to prevent pathogen entry into the plant cell.

Moreover, positive increases in the expression of *OsNAC103* (Os07G0683200), *OsNAC58* (Os03G0327800), *OsNAC4* (Os01G0816100), and *OsNAC131* (Os12G0123700) were observed in CBB23 vs. JG30 at different time intervals of post inoculation of PXO99^A^. The previous literature suggests that *OsNAC4* plays role in the hypersensitivity reaction (HR) cell death, accompanied by loss of plasma membrane integrity and typical morphological changes [56]. Whereas, *OsNAC58* was reported to be important in rice resistance to bacterial blight by activating the defense-related genes and ET/JA signaling-related genes [57].

The KEGG analysis exhibited that a cluster of DEGs, after PXO99^A^ infection, was enriched in the phenylpropanoid pathway. Previous literature reported that the phenylpropanoid pathway was involved in partial resistance to *M*. *oryzae* [58]. In *Arabidopsis* and populus, the phenylpropanoid pathway is regulated in stress conditions, and it is involved in the lignification process [59]. Furthermore, lignin formation is a key process for the protection of host plant under both abiotic and biotic stresses [60,61].

Plant hormones, JA, SA, and ET play important roles in regulating the plant development in response to various abiotic and biotic stresses [62]. In our experiment, SA-related genes (Os05G0102000 and Os11G0256900) were generally repressed after PXO99^A^ infection in CBB23 compared with JG30, and 14 ET-encoding DEGs were upregulated in CBB23 vs. JG30. Previous reports mentioned that SA was downregulated in *Xa7*-mediated resistance against bacterial blight at high temperature [63]. It has been found that ET is induced during compatible reaction, but not the incompatible reaction with *M. oryzae* [64]; additionally, the elevated ET production coincided with the appearance of HR and induction of the defense-related genes [65]. 

In HR type immunity, effector protein is released by the pathogen, to promote the disease in the host plant. Meanwhile, plants secrete proteases in a rapid and robust manner to lower the pathogenicity level [66,67]. We identified 13 different upregulated proteolysis responsive genes in CBB23 compared with JG30. In *Arabidopsis*, AvrPphB and AvrRpt2 were reported to be secreted in response to the *P. syringae* pv. *phaseolicola* and *P. syringae* pv. *tomato*, respectively [68,69].

The RNA-Seq data analysis at different time points in CBB23 vs. JG30 led to the identification of differentially expressed genes. Further detailed analyses are needed to generate innovative information regarding the pattern of resistance conferred by *Xa23*. Besides, these DEGs could be used for further experiments to exploit the disease resistance mechanism in rice plants.

## 4. Materials and Methods

### 4.1. Plant Material and Growth Conditions

Two *indica* rice genotypes, CBB23 (resistant) and JG30 (susceptible), were used for the experiment. These two genotypes are near-isogenic lines (identical background except a single gene of *Xa23*). The resistance variety, CBB23, was developed after four generations of backcrossing between *O*. *rufipogon* and susceptible *indica* variety, JG30 [15]. Initially, rice seeds were surface-sterilized in 70% ethanol for 5 min, rinsed with deionized water. Afterwards, these sterilized rice seeds were water-soaked overnight. After pre-germination, rice seeds were sown in pots and kept in the greenhouse of Institute of Crop Sciences, Chinese Academy of Agricultural Sciences (CAAS), Beijing, P.R. China. The condition of greenhouse in which rice seeds were grown was 25/30 °C under a 14 h light/10 h dark cycle with 80% relative humidity. 

### 4.2. Inoculation of Rice Plants with PXO99^A^ and Collection of Leaf Samples

The *Xoo* strain PXO99^A^ was used for inoculation into CBB23 and JG30 leaves. First, PXO99^A^ was subcultured on TSA plate (tryptophan 10 g/L; sucrose 10 g/L; glutamic acid 1 g/L, and agar 5 g/200 mL) for 48 h at 28 °C. Inoculum was prepared by suspending the bacterial culture in sterile water to obtain an OD_600_ of 1.0. Sixty day old plants (vegetative phase) were used for inoculation with PXO99^A^ through a needleless syringe. Inoculated leaves were harvested with three biological replicates at different time intervals (12 hpi, 24 hpi, 36 hpi, and 48 hpi, respectively) and immediately frozen in liquid nitrogen and stored at −80 °C until RNA extraction. Here, mock leaves (without inoculation) of CBB23 and JG30 were denoted as C0 and J0, respectively.

Meanwhile, the leaves of CBB23 and JG30 were infected with PXO99^A^ by using scissors dipped in bacterial suspensions to clip leaves 1–2 cm down from the tip of the leaf blade. After 2 weeks post inoculation, lesion length was measured from the cut surface to the distal-most position of the leaf blade that exhibited chlorotic or water-soaked lesions.

### 4.3. RNA Extraction and Library Preparation for Illumina Sequencing

All PXO99^A^ infected and mock leaves were ground into a fine powder in liquid nitrogen by constant crushing using sterilized and chilled pestle and mortar, to isolate RNA by using TRIzol Reagent kit (TIANGEN, Beijing, China) according to the manufacturer’s instruction. Thereafter, the samples were purified by using RNase-free DNase I (TaKaRa, Kyoto, Japan) to remove the genomic DNA. Total concentration of RNA was determined using NanoDrop microvolume spectrophotometer (Thermo Scientific NanoDrop Products, Waltham, MA, USA). Afterwards, Illumina HiSeq2500 platform was used for RNA-Seq analysis. Library construction and RNA-Seq were carried out by Novogene Bioinformatics Technology Co., Ltd., Beijing, China.

### 4.4. Data Analysis

Initially, read quality of the raw data was evaluated by the fastQC application v0.11.2 [70]. Each paired-end library had insert size between 200–300 bp. The preprocessing of the data was performed by removing adapter sequences and low quality reads by cutadapt tool [71]. Thereafter, clean sequence reads were mapped to the available Japonica rice Nipponbare genome (http://rapdb.dna.affrc.go.jp/download/irgsp1.html) [72] using TopHat v2.0.12 (http://ccb.jhu.edu/software/tophat/index.shtml) [73], by applying Bowtie2 v2.0.0 [74]. Afterwards, Cufflinks was applied to measure the transcript abundance and gave the expression of each transcript, in FPKM (fragments per kilobase pair of exon model per million fragments mapped) [75]. The differential expression of the transcripts was calculated by using the ratio of two different samples FPKM values of a single gene by Cuffdiff. Actually, Cuffdiff is a tool for comparing expression level of transcripts and tells which genes are up- or downregulated between two or more conditions. Afterwards, the subsequent list of DEGs was filtered with Log_2_FC ≥1 (upregulated genes) or ≤−1 (downregulated genes).

### 4.5. Gene Enrichment Analysis

We conducted GO analysis to describe the characteristics and reaction features of the DEGs that were retrieved at different time periods before and after PXO99^A^ infection from the NILs. The GO analysis was carried out by AgriGO software [76]. GO terms with FDR value ≤0.05 were considered significantly enriched by DEGs. For pathway analysis, we mapped all the DEGs in terms of KEGG and retrieved the significantly enriched pathway with *p*-value ≤ 0.05 [77]. For graphical overview of the biotic stress response, we used MapMan tool (http://MapMan.gabipd.org) [78].

### 4.6. Validation of RNA-Seq Data

Different genes from DEGs were selected to validate the RNA-Seq data. The corresponding sequences of the selected genes were retrieved from rice annotation project database. Primers of the DEGs were designed according to the transcript sequences by using AmplifX 1.5.4 software [79], and primers used in the experiment are listed in Table S9. Moreover, the total leaf RNA was isolated from mock and PXO99^A^ infected leaves of CBB23 and JG30 by TRIzol reagent, and the purified RNA was reverse-transcribed through cDNA synthesis kit (TransGen, Beijing, China) according to the manufacturer’s instructions. Quantitative real-time PCR (qRT-PCR) was performed in 96-well plates on an ABI prism 7500 Real-Time PCR system (Applied Biosystem, Foster City, CA, USA) using SYBR Green Master ROX (TaKaRa). Ubiquitin was used as an internal control during qRT-PCR. The thermal cycler conditions were 95 °C for 30 s, followed by 40 cycles of 95 °C for 10 s, 60 °C for 34 s and 72 °C for 15 s. The relative expression level of the selected genes was calculated with the 2^−ΔΔ*C*T^ method [80].

## Figures and Tables

**Table 1:** An overview of sequencing and assembly of CBB23 and JG30 samples.

Samples	Raw Reads	Clean Reads	Total Mapped	Q Value (30%)	GC %
C0	46,594,244	44,372,944	36,489,861 (82.23%)	91	54
J0	51,085,434	48,289,010	39,611,914 (82.03%)	91	54
CBB23-12 hpi	57,398,598	54,774,152	45,086,818 (82.31%)	92	54
CBB23-24 hpi	52,122,832	49,294,374	41,275,771 (83.73%)	91	55
CBB23-36 hpi	65,574,380	62,882,740	50,963,164 (81.04%)	92	53
CBB23-48 hpi	41,893,788	40,539,554	33,922,512 (83.68%)	92	54
JG30-12 hpi	49,890,010	48,749,132	40,250,051 (82.57%)	93	55
JG30-24 hpi	54,891,574	52,116,660	43,303,751 (83.09%)	91	55
JG30-36 hpi	45,507,160	44,119,566	35,292,193 (79.99%)	92	53
JG30-48 hpi	57,418,698	54,514,722	45,769,331 (83.96%)	91	55

## Supplementary Materials

The following are available online at http://www.mdpi.com/1422-0067/19/3/717/s1.

## Availability of Supporting Data

All the original RNA-Seq data has been submitted to the NCBI Sequence Read Archive under the accession number of SRP132257.
